# *Trans* Fatty Acid Intakes and Food Sources in the U.S. Population: NHANES 1999–2002

**DOI:** 10.1007/s11745-012-3704-z

**Published:** 2012-08-18

**Authors:** Penny M. Kris-Etherton, Michael Lefevre, Ronald P. Mensink, Barbara Petersen, Jennifer Fleming, Brent D. Flickinger

**Affiliations:** 1 Department of Nutritional Sciences, 319 Chandlee Laboratory, The Pennsylvania State University, University Park, PA 16802 USA; 2 Department of Nutrition, Dietetics and Food Science, Utah State University, 9815 Old Main Hill, Logan, UT 84322-9815 USA; 3 Department of Human Biology, Nutrition and Toxicology Research Institute Maastricht, Maastricht University, P.O. Box 616, 6200 MD Maastricht, The Netherlands; 4 Exponent, Inc., 1150 Connecticut Ave NW, Suite 1100, Washington, DC 20036 USA; 5 Archer Daniels Midland Company, Randall Research Center, 1001 N. Brush College Rd., Decatur, IL 62521 USA

**Keywords:** *Trans* fatty acid intake, Industrial *trans* fatty acid intake, Fatty acid intake, Quintiles of *trans* fatty acid intake, Food sources of *trans* fatty acids, NHANES 1999–2002

## Abstract

Because of efforts to decrease *trans* fatty acids (TFA) in the food supply, intake should be assessed in the population to establish a baseline TFA intake. The 1999–2002 National Health and Nutrition Examination Survey (NHANES) was used to identify a benchmark for TFA intake. TFA was estimated by mean, median, and quintile of intake, TFA intake data were weighted using the NHANES 4-year sample weights. The main outcome measures included TFA intake in grams per day and percentage of energy in the top 25 food sources of TFA. Data are reported for 16,669 individuals ≥3 years of age. Median TFA intake was 2.3 % of calories (5 g/day) with 0.9–4.5 % of energy (1.5–13.1 g/day) over different quintiles of intake. Mean TFA intake was 2.5 % of energy (6.1 g/day). The range of TFA intake in the fifth quintile was very large, i.e., 3.5–12.5 % of energy or 8.8–92.4 g/day. Increasing quintiles of TFA intake were associated with increases in total fat (26.7–37.6 % of energy), saturated fat (7.6–10.5 % of energy), and calories (for those >20 years of age: 2,416–2,583 for men and 1,679–1,886 for women). Major food sources of dietary TFA were cakes, cookies, pies, and pastries. Based on current dietary guidance to consume as little industrial TFA as possible, much progress is needed to attain this goal, including food industry efforts to remove TFA from the food supply and educating the public about making healthy food choices.

## Introduction

Dietary recommendations have been made in the United States and globally to decrease *trans* fatty acids (TFA) produced by partial hydrogenation of vegetable oils [1, 2]. These recommendations are based on numerous studies demonstrating many adverse health effects of TFA [3]. Multiple strategies have been invoked in the United States to decrease dietary TFA, including nutrition labeling of TFA on the Nutrition Facts Panel (law passed in 2003; enacted in 2006), as well as passage of legislation banning or restricting use of fats and oils containing TFA in some states and cities [4, 5]. In 2009, legislation to limit TFA in the food supply was enacted in 1 state and proposed in 13 states and the District of Columbia [6]. Based on the success of this approach in New York, it seems that legislative strategies may be an effective strategy for decreasing TFA in foods [7]. Mozaffarian and Stampfer [7] estimated that a 1 % reduction in TFA intake could prevent 72,000 cardiovascular disease-related deaths per year. The U.S. Food and Drug Administration (FDA) have also suggested that the removal of TFA from just 3 % of breads and cakes and 15 % of cookies and crackers would save up to US $59 billion in health care costs over the next 20 years [8]. Consequently, the food industry is actively lowering TFA in the food supply by developing new fats and oils and modifying existing ones [9, 10].

The last comprehensive analysis of TFA intake was published by Allison et al. [11] in 1999, using 1989–1991 data collected from the U.S. Department of Agriculture’s (USDA) Continuing Survey of Food Intakes by Individuals (CSFII). At that time, the average TFA intake in the United States was 2.6 % of calories, or 5.3 g/day [11]. Since the work by Allison et al. was published, many food products have had TFA removed and/or reduced appreciably [5, 12]. Much of this has happened as the result of legislation (announced on January 9, 2003) mandating TFA labeling on the Nutrition Facts Panel by January 1, 2006. Clearly, there is a need to evaluate the effects of this announcement and legislation on current TFA intake, and this needs to be done in the context of the most current TFA intake prior to the FDA’s announcement. Consequently, it is important to benchmark current TFA intake against intake that is more recent than the 1989–1991 data to evaluate the impact of legislation enacted to decrease TFA intake.

The purpose of this study was to assess TFA intake in different population groups in the United States using more recent intake data from the 1999–2002 National Health and Nutrition Examination Survey (NHANES) just prior to the time that legislation was passed for TFA to be listed on the Nutrition Facts Panel. We also evaluated food sources of TFA in different population groups. A better understanding of the major food sources of TFA would be helpful for ongoing efforts to markedly lower TFA in the food supply.

## Materials and Methods

### Estimation of TFA Intake During 1999–2002

To estimate dietary intake of TFA, we used 1999–2002 NHANES consumption data as well as the Foods Analysis and Residue Evaluation Program (FARE) software (v.7.997; Exponent, Washington, DC). FARE is proprietary data processing software that was developed to facilitate the mining of the thousands of individual intake records in the NHANES database. A more detailed description of the analysis done by using the FARE program is described by DiRienzo et al. [13]. FARE is used by the FDA, the USDA, the California Office of Environmental Health Hazard Assessment, and the Health Canada Pest Management Regulatory Agency to derive estimates of food, nutrient, and contaminant intakes based on NHANES and CSFII consumption data.

Dietary assessment in NHANES 1999–2002 was conducted using two 24-h dietary recalls, one in person and a second one by telephone [14]. The TFA content of foods was derived from the NHANES dataset. The NHANES used the *Trans* Fatty Acid Database (laboratory analyses completed between 1990 and 1993) that was released in 1995 as “Special table 1: Fat and fatty acid content of selected foods containing *trans* fatty acids.” This is referred to as the USDA Nutrient Database for Standard Reference (Release 15) which was used to map the *trans* fat content of analyzed foods to the foods reported in the 1999–2002 NHANES. Total dietary TFA intakes were calculated by multiplying the amount of food consumed by the concentration of TFA in that food, and were expressed as a percentage of individual energy intakes per day. The TFA intake data were weighted using the NHANES 4-year sample weights as a means to ensure that the results are representative of the US population (aged ≥3 years). Two separate analyses were required to determine the quintile cutoff points for grams per day as well as percentage of energy. The quintiles for the analyses expressed as grams per person per day were calculated based upon grams per day of TFA intake. A second set of population quintile groups was calculated for the percentage of energy analyses based upon the per capita TFA intake expressed as a percentage of energy. The values provided within each quintile are the mean nutrient intakes of that quintile of the population. The inter-quintile ranges of TFA intake from the total diet for each quintile are provided. The un-weighted sample sizes are not exactly the same for each quintile; however, the quintiles are balanced after applying the statistical weights. Populations were defined by age and gender according to the criteria used by Allison et al. [11] so that results could be compared.

### Estimation of Food Sources of TFA

Twenty-five food categories were established based on pre-established food groupings in NHANES (e.g., grains and salty snacks, milk and milk products, etc.) and then further sub-divided to allow identification of categories of foods as eaten (e.g., ready-to-eat breakfast cereals) or that were mentioned in previous studies to be contributors to TFA intakes. The amount of TFA in food (g/100 g) was derived from NHANES recipes as described above.

## Results

**Fig. 1:**
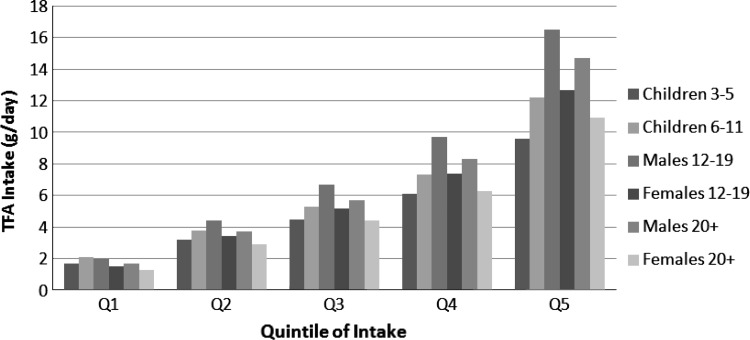
Dietary TFA (g/day) in all demographic groups by quintile of intake

**Fig. 2:**
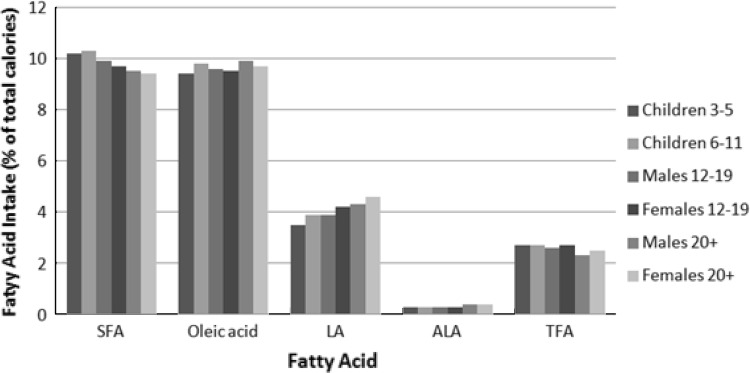
Fatty acid intake (% of calories) by demographic group. *SFA* saturated fatty acid, *LA* linoleic acid, *ALA* alpha-linolenic acid, *TFA* *trans* fatty acid

The mean, median, 90th percentile, and quintiles of TFA intake, as well as other fatty acids, total fat (in grams per day), and energy in the U.S. population (aged ≥3 years), are reported for 16,669 subjects (Table 1; Fig. 1) as well as percentages of energy (Table 2). It is apparent that males aged 12–19 years have the highest mean TFA intake expressed as grams per day compared with the other populations for each quintile. When expressed as a percentage of energy, the intake of TFA was similar in all population groups. Subjects were stratified on the basis of total TFA intake as grams per day or percentage of energy, thereby explaining why the number of subjects differs within a quintile between tables for all population groups. The median TFA intake between 1999 and 2002 was 2.3 % of calories (5 g/day) with a range of 0.9–4.5 % of energy (1.5–13.1 g/day) over the different quintiles of intake. The mean TFA intake was 2.5 % of energy (6.1 g/day). There was a remarkable range of TFA intake in the top quintile (3.5–12.5 % of energy, or 8.8–92.4 g/day). Increasing quintiles of TFA intake were associated with increases in total fat (range, 26.7–37.6 % of energy), saturated fat (range, 7.6–10.5 % of energy), as well as calories (for men and women aged >20 years: 2,416–2,583 for men and 1,679–1,886 for women). Interestingly, intakes of *cis*-monounsaturated fatty acid (MUFA) and linoleic acid (both as grams per day and percentage of energy) also increased with increasing TFA intake. Notably, from the first to the fifth quintile, energy intakes increased approximately twofold in each cohort, whereas TFA increased 5- to 8-fold across the quintiles for all cohorts. On a percentage-of-energy basis, TFA increased about 4.5- to 5-fold across the quintiles for all cohorts. This suggests that food choices varied and dietary patterns differed across the TFA quintiles. Likewise, as a percentage of energy, TFA intake also increased concomitant with increases in percentage of energy from total fat, saturated fatty acids (SFA), MUFA, and polyunsaturated fatty acids, with the increases in TFA being proportionally greater. Specifically, TFA increased 4- to 5-fold across quintiles of TFA intake, whereas energy intake increased from 7 to 17 %. Trends for TFA intakes were similar for all population groups studied (Tables 1, 2), with median TFA intake ranging from 2.1 to 2.5 % of calories (4.4–6.6 g/day). The mean TFA intake ranged from 2.3 to 2.7 % of calories (5.0–7.8 g/day). Intakes of TFA (as a percentage of calories), as well as SFA, oleic acid, linoleic acid, and alpha-linolenic acid, were similar among the different cohorts studied (Fig. 2).

**Table 1:** Per capita total dietary intakes of *trans* fatty acids, energy, fat, and select fatty acids (g/day)^a^

Population, energy, and nutrient	Mean	Median	90th percentile	Q1^b^	Q2	Q3	Q4	Q5
Children aged 3–5 years								
Participants (*n*)	1,034			220	206	216	198	194
IQR				0–2.50	2.50–3.75	3.75–5.28	5.28–7.24	7.24–35.13
Total *trans* fatty acids	5.0	4.5	8.9	1.7	3.2	4.5	6.1	9.6
Energy (kcal)^c^	1,650	1,591	2,360	1,130	1,438	1,644	1,867	2,169
Total fat	59.6	56.5	91.4	36.3	48.6	59.1	69.7	84.1
SFA (14:0, 6:0,18:0)	18.8	17.8	30.6	11.5	16.0	19.0	21.9	25.8
14:0	2.1	1.9	3.9	1.4	2.0	2.2	2.4	2.6
16:0	11.1	10.5	17.9	6.8	9.4	11.2	13.0	15.1
18:0	5.6	5.3	9.2	3.2	4.5	5.6	6.5	8.1
18:1c	17.3	16.2	28.0	10.5	13.6	16.9	20.4	24.9
18:2c	6.3	5.5	11.1	3.8	5.1	6.4	7.5	8.7
18:3c	0.5	0.4	0.9	0.4	0.4	0.5	0.6	0.7
Children aged 6–11 years								
Participants (*n*)	2,124			444	432	368	416	464
IQR				0–3.04	3.04–4.60	4.60–6.10	6.10–8.60	8.60–35.01
Total *trans* fatty acids	6.1	5.2	10.8	2.1	3.8	5.3	7.3	12.2
Energy (kcal)^c^	1,996	1,883	2,952	1,427	1,717	1,864	2,214	2,757
Total fat	73.9	68.4	116.1	46.4	60.6	69.6	82.7	110.4
Total SFA	22.9	21.2	37.1	14.9	19.2	21.9	26.6	31.8
14:0	2.4	2.1	4.3	1.7	2.1	2.5	3.0	2.7
16:0	13.6	12.6	21.9	9.1	11.5	13.0	15.7	18.9
18:0	6.9	6.3	11.3	4.2	5.6	6.4	8.0	10.3
18:1c	22.0	20.2	34.9	13.9	18.0	20.0	24.3	33.6
18:2c	8.7	7.1	15.6	5.6	7.0	8.0	9.1	14.0
18:3c	0.7	0.5	1.3	0.5	0.5	0.6	0.7	1.0
Males aged 12–19 years								
Participants (*n*)	2,252			480	477	467	429	399
IQR				0–3.28	3.28–5.49	5.49–8.06	8.06–11.79	11.79–92.41
Total *trans* fatty acids	7.8	6.6	14.7	2.0	4.4	6.7	9.7	16.5
Energy (kcal)^c^	2,672	2,484	4,238	1,702	2,075	2,633	3,078	3,872
Total fat	95.8	87.1	162.1	44.6	72.0	93.4	115.5	149.2
SFA (14:0, 16:0, 18:0)	29.7	26.8	51.4	15.6	23.0	29.5	36.0	44.5
14:0	3.0	2.5	6.0	1.7	2.5	3.0	3.7	4.1
16:0	17.8	16.0	31.1	9.5	13.9	17.7	21.4	26.3
18:0	9.0	8.2	15.6	4.4	6.6	8.8	10.9	14.1
18:1c	28.9	25.6	49.0	14.7	21.6	28.2	34.8	45.0
18:2c	11.6	9.8	21.5	5.9	9.1	11.8	13.3	18.0
18:3c	0.9	0.7	1.7	0.5	0.7	0.9	1.1	1.4
Females aged 12–19 years								
Participants (*n*)	2,263			473	443	445	453	449
IQR				0–2.59	2.59–4.24	4.24–6.06	6.06–8.94	8.94–36.39
Total *trans* fatty acids	6.1	5.2	11.7	1.5	3.4	5.2	7.4	12.7
Energy (kcal)^c^	1,987	1,882	3,095	1,268	1,692	1,910	2,293	2,761
Total fat	71.5	65.7	119.4	36.6	55.4	67.6	86.6	110.8
SFA (14:0, 16:0, 18:0)	21.7	19.6	38.4	11.4	17.7	20.7	26.5	32.1
14:0	2.2	1.8	4.5	1.2	1.9	2.2	2.8	2.9
16:0	13.0	11.7	22.9	7.0	10.6	12.4	15.7	18.9
18:0	6.6	6.0	11.6	3.2	5.1	6.1	8.0	10.2
18:1c	21.1	19.2	35.5	11.1	16.2	19.8	25.1	33.0
18:2c	9.3	7.5	18.4	5.3	7.1	8.8	11.0	14.2
18:3c	0.8	0.6	1.5	0.5	0.6	0.8	0.8	1.1
Males aged ≥20 years								
Participants (*n*)	4,236			967	963	878	753	675
IQR				0–2.75	2.75–4.64	4.64–7.01	7.01–10.20	10.20–59.78
Total *trans* fatty acids	6.8	5.6	13.1	1.7	3.7	5.7	8.3	14.7
Energy (kcal)^c^	2,596	2,440	3,959	1,772	2,178	2,505	2,874	3,647
Total fat	95.8	86.7	161.4	51.6	74.1	91.5	109.2	152.6
SFA (14:0, 16:0, 18:0)	28.0	25.0	50.0	14.4	21.8	27.6	32.2	44.2
14:0	2.6	2.1	5.3	1.3	2.1	2.7	3.0	3.9
16:0	16.9	15.0	29.8	8.9	13.2	16.6	19.4	26.3
18:0	8.5	7.5	15.1	4.1	6.4	8.3	9.8	14.0
18:1c	28.8	25.9	49.9	15.6	21.9	27.5	32.9	45.8
18:2c	12.3	10.2	23.4	6.7	9.2	11.5	14.4	19.4
18:3c	1.1	0.8	2.2	0.7	0.8	1.0	1.2	1.6
Females aged ≥20 years								
Participants (*n*)	4,760			1,039	1,007	953	873	888
IQR				0–2.15	2.15–3.62	3.62–5.25	5.25–7.56	7.56–35.0
Total *trans* fatty acids	5.2	4.4	9.8	1.3	2.9	4.4	6.3	10.9
Energy (kcal)^c^	1,838	1,736	2,830	1,243	1,590	1,768	2,010	2,579
Total fat	68.4	61.8	116.2	37.8	53.8	65.6	78.3	106.4
SFA (14:0, 16:0, 18:0)	19.5	17.4	34.6	10.3	15.7	18.9	22.6	30.0
14:0	1.9	1.5	3.8	1.0	1.6	1.8	2.2	2.6
16:0	11.7	10.5	20.9	6.3	9.5	11.4	13.5	17.9
18:0	5.9	5.3	10.4	2.9	4.6	5.7	6.8	9.6
18:1c	20.1	18.0	34.8	11.0	15.5	19.2	22.8	31.7
18:2c	9.4	7.7	18.0	5.3	7.1	9.1	10.8	14.6
18:3c	0.8	0.6	1.7	0.5	0.7	0.8	0.9	1.2

^a^Per capita estimates are based on food consumption data for all individuals who responded during the one NHANES survey day. This analysis includes all foods in the diet. Conducted in FARE version 7.997

^b^Quintiles calculated based upon per capita total *trans* fatty acid consumption. Number of participants indicates the unweighted sample size, interquintile range (*IQR*) indicates cutoff values of trans fat intake, and mean intake values are reported within each quintile

^c^Energy units are kcal/day

**Table 2:** Per capita total dietary intakes of *trans* fatty acids, fat, select fatty acids, and energy (% total energy)^a^

Population, nutrient	Mean	Median	90th percentile	Q1^b^	Q2	Q3	Q4	Q5
Children aged 3–5 years								
Participants (*n*)	1,034			231	218	186	210	189
IQR				0–1.65	1.65–2.31	2.31–2.79	2.79–3.61	3.61–8.83
Total *trans* fatty acids	2.7	2.5	4.4	1.2	2.0	2.5	3.1	4.6
Total fat	32.4	32.0	42.1	28.1	31.3	32.5	33.9	35.9
Sum 14:0, 16:0, 18:0	10.2	10.0	14.4	9.1	10.3	10.5	10.9	10.2
14:0	1.2	1.1	2.0	1.2	1.3	1.2	1.2	0.9
16:0	6.0	5.9	8.4	5.4	6.1	6.2	6.4	6.0
18:0	3.0	3.0	4.2	2.5	2.9	3.1	3.3	3.3
18:1c	9.4	9.2	13.0	8.1	8.9	9.2	10.0	10.7
18:2c	3.5	3.1	5.6	2.8	3.5	3.4	3.7	3.8
18:3c	0.3	0.2	0.5	0.3	0.3	0.3	0.3	0.3
Energy (kcal)^c^	1,650	1,591	2,360	1,525	1,604	1,742	1,668	1,703
Children aged 6–11 years								
Participants (*n*)	2,124			435	433	402	432	422
IQR				0–1.64	1.64–2.26	2.26–2.88	2.88–3.73	3.73–8.54
Total *trans* fatty acids	2.7	2.5	4.4	1.2	2.0	2.6	3.3	4.7
Total fat	33.1	33.2	42.9	29.9	31.6	33.2	34.1	36.6
Total SFA	10.3	10.1	14.2	9.6	10.2	10.4	10.5	10.7
14:0	1.1	1.0	1.8	1.1	1.2	1.1	1.0	1.0
16:0	6.1	6.1	8.5	5.8	6.0	6.2	6.2	6.3
18:0	3.1	3.1	4.3	2.7	2.9	3.1	3.2	3.4
18:1c	9.8	9.6	13.6	8.9	9.1	10.0	10.2	11.0
18:2c	3.9	3.4	6.4	3.6	3.6	4.0	4.1	4.3
18:3c	0.3	0.3	0.5	0.3	0.3	0.3	0.3	0.3
Energy (kcal)^c^	1,996	1,883	2,952	1,806	2,067	2,057	2,058	1,989
Males aged 12–19 years								
Participants (*n*)	2,252			454	445	443	446	464
IQR				0–1.47	1.47–2.09	2.09–2.76	2.76–3.62	3.62–9.90
Total *trans* fatty acids	2.6	2.4	4.4	1.0	1.8	2.4	3.2	4.7
Total fat	32.0	32.5	42.3	25.8	31.1	32.3	34.7	36.2
Sum 14:0, 16:0, 18:0	9.9	9.9	14.0	8.3	10.3	10.2	10.6	10.2
14:0	1.0	0.9	1.8	0.9	1.1	1.0	1.1	0.8
16:0	5.9	5.9	8.3	5.0	6.2	6.1	6.3	6.1
18:0	3.0	3.0	4.1	2.3	3.0	3.0	3.2	3.3
18:1c	9.6	9.7	13.3	7.7	9.4	9.7	10.3	11.1
18:2c	3.9	3.5	6.5	3.2	3.6	4.0	4.3	4.3
18:3c	0.3	0.3	0.6	0.3	0.3	0.3	0.3	0.3
Energy (kcal)^c^	2,672	2,484	4,238	2,390	2,740	2,793	2,727	2,704
Females 12–19 years								
Participants (*n*)	2,263			457	431	443	485	447
IQR				0–1.49	1.49–2.10	2.10–2.80	2.80–3.88	3.88–12.46
Total *trans* fatty acids	2.7	2.5	4.7	1.0	1.8	2.4	3.3	5.0
Total fat	32.0	32.0	43.6	25.9	30.6	32.3	34.2	37.2
Sum 14:0, 16:0, 18:0	9.7	9.6	14.1	8.2	9.8	10.0	10.3	10.2
14:0	1.0	0.9	1.8	0.9	1.1	1.1	1.0	0.8
16:0	5.8	5.7	8.3	5.0	5.9	6.0	6.1	6.1
18:0	2.9	2.9	4.3	2.3	2.8	3.0	3.2	3.3
18:1c	9.5	9.4	13.3	7.7	9.0	9.3	10.2	11.2
18:2c	4.2	3.6	7.0	3.7	4.0	4.1	4.5	4.7
18:3c	0.3	0.3	0.6	0.3	0.3	0.3	0.3	0.4
Energy (kcal)^c^	1,987	1,882	3,095	1,746	1,952	2,099	2,095	2,045
Males aged ≥20 years								
Participants (*n*)	4,236			837	918	864	825	792
IQR				0–1.21	1.21–1.82	1.82–2.46	2.46–3.37	3.37–10.51
Total *trans* fatty acids	2.3	2.1	4.1	0.8	1.5	2.1	2.9	4.4
Total fat	32.8	32.7	45.0	26.2	31.31	33.19	35.1	38.1
Sum 14:0, 16:0, 18:0	9.5	9.5	14.1	7.3	9.4	10.0	10.3	10.6
14:0	0.9	0.8	1.6	0.7	0.9	1.0	0.9	0.8
16:0	5.8	5.8	8.4	4.5	5.7	6.0	6.2	6.3
18:0	2.9	2.9	4.3	2.1	2.8	3.0	3.2	3.5
18:1c	9.9	9.7	14.3	7.9	9.5	10.0	10.6	11.6
18:2c	4.3	3.7	7.2	3.5	3.9	4.2	4.5	5.1
18:3c	0.4	0.3	0.7	0.3	0.4	0.4	0.4	0.4
Energy (kcal)^c^	2,596	2,440	3,959	2,416	2,605	2,603	2,772	2,583
Females aged ≥20 years								
Participants (*n*)	4,760			962	1,013	968	926	891
IQR				0–1.31	1.31–1.98	1.98–2.66	2.66–3.52	3.52–10.00
Total *trans* fatty acids	2.5	2.3	4.3	0.9	1.7	2.3	3.0	4.5
Total fat	33.0	33.0	45.5	26.6	31.8	33.2	35.4	38.2
Sum 14:0, 16:0, 18:0	9.4	9.2	14.1	7.3	9.3	9.8	10.2	10.4
14:0	0.9	0.8	1.7	0.7	1.0	1.0	1.0	0.8
16:0	5.7	5.5	8.4	4.5	5.6	5.9	6.1	6.2
18:0	2.8	2.8	4.2	2.1	2.7	2.9	3.1	3.4
18:1c	9.7	9.6	14.1	7.7	9.2	9.8	10.4	11.5
18:2c	4.6	4.0	8.2	3.6	4.3	4.5	5.1	5.3
18:3c	0.4	0.3	0.8	0.4	0.4	0.4	0.4	0.4
Energy (kcal)^c^	1,838	1,736	2,830	1,679	1,816	1,893	1,918	1,886

^a^Per capita estimates are based on food consumption data for all individuals who responded during the one NHANES survey day, whether they reported eating the food(s) or ingredient(s) of interest. Percentage of energy for total fat and the individual fatty acids should be determined per individual and grouped within quintiles based on *trans* intake as %en. Conducted in FARE version 7.99

^b^Quintiles calculated based upon % energy from total *trans* fatty acid consumption. Interquintile range (*IQR*) indicates cutoff values of *trans* fat intake, and mean intake values are reported within each quintile

^c^Energy units are kcal/day. The values for each quintile are the mean kcal/day consumed by the participants who consumed the amount *trans* fat as a % of energy listed in the interquintile range

**Fig. 3:**
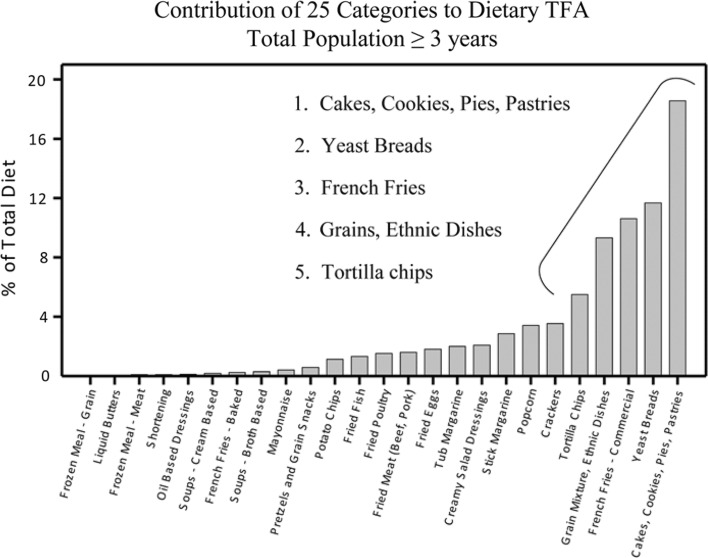
Contribution of 25 food categories to dietary TFA. The *Y* axis represents the percentage of total TFA contributed by the respective food category

The top 25 food categories that accounted for 79 % of TFA intake in the United States were identified on the basis of gram quantity of TFA consumed (Fig. 3). In our analysis, the major source of dietary TFA in the total population aged ≥3 years was cakes, cookies, pies, and pastries (Fig. 3), accounting for approximately 19 % of total TFA intake. Importantly, this food source of TFA was approximately 2-fold higher than the next three leading sources, which include yeast breads, French fries (commercial), and grain mixtures/ethnic mixed dishes. The fifth highest contributor was tortilla chips (accounting for 5.5 % of total TFA). Stick margarine provided 2.9 % of total TFA, and was ranked eighth in TFA intake. The 10 major food sources of TFA across the different population cohorts were very similar (Table 3).

**Table 3:** Top 10 food group contributors to *trans* fat intake in different population groups

All, ≥3 years	Children, 3–5 years	Children, 6–11 years	Males, 12–19 years	Females, 12–19 years	Males, ≥20 years	Females, ≥20 years
Cakes, cookies, pies, pastries	Cakes, cookies, pies, pastries	Cakes, cookies, pies, pastries	Cakes, cookies, pies, pastries	Cakes, cookies, pies, pastries	Cakes, cookies, pies, pastries	Cakes, cookies, pies, pastries
Yeast breads	French fries	Grains	French fries	French fries	Yeast breads	Yeast breads
French fries	Grains	French fries	Grains	Grains	French fries	French fries
Grains	Yeast breads	Yeast breads	Yeast breads	Yeast breads	Grains	Grains
Tortilla chips	Tortilla chips	Tortilla chips	Tortilla chips	Tortilla chips	Tortilla chips	Crackers
Crackers	Crackers	Crackers	Popcorn	Crackers	Stick margarine	Tortilla chips
Popcorn	Popcorn	Popcorn	Crackers	Popcorn	Popcorn	Popcorn
Stick margarine	Stick margarine	Stick margarine	Fried meat (beef, pork)	Stick margarine	Crackers	Stick margarine
Creamy salad dressing	Tub margarine	Tub margarine	Stick margarine	Creamy salad dressing	Fried eggs	Creamy salad dressing
Tub margarine	Fried eggs	White potatoes	Fried poultry	Fried poultry	Fried meat (beef, pork)	Tub margarine

A comparison of the above data with the 1989–1991 CSFII data [11] showed that the mean and median values were similar across cohorts, whereas TFA intake at the 90th percentile was higher (Table 4) in our analysis. In terms of the percentage of calories from TFA in 1989–1991, all cohorts in the 90th percentile of TFA intake consumed approximately 3.2 % of calories. In contrast, using the 1999–2002 NHANES data, TFA intake was 4.1–4.7 % of calories in similar cohorts. These results clearly indicate that consumption of TFA food sources increased considerably between 1991 and 2002 in individuals in the 90th percentile of TFA intake.

**Table 4:** Comparison of *trans* fatty acid intake in 1989–1991 and 1999–2002 (g/day)

Population	CSFII^a^ (1989–1991)	NHANES (1999–2002)
Children, 3–5 years		
Mean	4.1	5.0
Median	3.9	4.5
90th percentile	6.6	8.9
Males, 12–19 years		
Mean	7.1	7.8
Median	6.7	6.6
90th percentile	11.4	14.7
Females, 12–19 years		
Mean	5.1	6.1
Median	4.9	5.2
90th percentile	8.6	11.7
Males 20–49 years, 50–69 years; males ≥20 years^a^		
Mean	6.6/5.8	6.8
Median	5.9/5.1	5.6
90th percentile	11.6/10.2	13.1
Females 20–49 years, 50–69 years; females ≥20 years^a^		
Mean	4.6/4.2	5.2
Median	4.2/3.7	4.4
90th percentile	8.0/7.4	9.8

^a^CSFII adult data were divided into subgroups of 20–49 and 50–69 years; NHANES reported all adults in one group (≥20 years)

## Discussion

The mean TFA intake in children and adults in the United States between 1999 and 2002 was 5.0–7.8 g/day (2.3–2.7 % of energy). In all cohorts, the range in TFA intake from the first quintile (1.3–2.0 g/day) to the fifth quintile (9.6–16.5 g/day) varied by approximately 5- to 8-fold. On a percentage-of-calories basis, TFA consumption ranged from 0.8–1.2 % (quintile 1) to 4.4–5.0 % of calories (quintile 5). For all population groups, there was remarkable variation in the range of TFA intake on a grams-per-day basis, as well as a percentage-of-energy basis in quintile 5. Variation was greatest in males aged 12–19 years, with intake that varied from 12 to 92 g/day in quintile 5. Collectively, these data illustrate that some individuals consume large quantities of TFA (in quintile 5), whereas there are others who eat little TFA (in quintile 1). Interestingly, as TFA intakes increased across quintiles, energy, total fat, and SFA intakes also increased in all populations. However, there were important differences in the magnitude of increases for each of these dietary constituents. Although there were noticeable increases in total fat and SFA intake across quintiles, as well as somewhat modest increases in energy intake across quintiles (specifically for the fatty acid data presented as percentages of energy in Table 2), the proportional increase in TFA was dramatically greater.

Collectively, our results indicate a change in dietary patterns across quintiles. Specifically, TFA-containing foods were either added to the diet or substituted for non-TFA-containing foods. Given that the proportional increase in dietary TFA was greater than the increase in energy, it seems that substitution of TFA-containing foods was more common. Thus, a high TFA intake reflects a different dietary pattern compared with a lower TFA intake. In fact, starting with quintile 2, it appears that the dietary patterns are shifting because of increases in the proportions of TFA, SFA, and total fat (as a percentage of energy) relative to the increase in energy intake. However, although all population groups showed similar changes in fatty acids and energy across quintiles, it can be concluded that dietary patterns within a quintile are likely comparable among the different population groups.

It is telling that the results of this study show that the population evaluated in 1999–2002 consumed similar amounts of TFA compared to the U.S. population surveyed in 1989–1991 [11]. In fact, the mean and median TFA intakes are similar in both studies. However, what is striking between the two studies is the increase in TFA intake in the 90th percentile. As Table 4 shows, there were approximately 20 and 27 % higher TFA intakes in men and women, respectively, in the 1999–2002 database versus the earlier survey. Collectively, this finding suggests that there is a relatively large cohort of individuals in the population who have made changes in their diet in a manner that is incongruent with current dietary recommendations. It is important to appreciate that the large gap between the 99.9th and 100th percentiles of TFA intake (34 vs. 92 g/day) most likely represents only a very small group of high-TFA consumers, which would be expected to slightly skew the results for this cohort. Nonetheless, these findings indicate that there are individuals who follow extreme dietary practices, putting them at high risk for chronic disease and malnutrition.

The food sources of dietary TFA are similar among the different population groups studied. The major TFA sources were cakes, cookies, pies, and pastries, as well as yeast breads, French fries, grains and ethnic dishes, and tortilla chips. It is important to note that many food sources of TFA are also major contributors of SFA [8], such as grain-based desserts, savory snacks, ethnic dishes, and French fries/fried potatoes. Many of these foods are typically classified as discretionary calories and consequently should be limited in the diet. Reducing these foods would not only decrease TFA but also SFA and excess calories.

Our analysis provides useful information about TFA intake and food sources in the United States prior to the onset of legislative action intended to decrease TFA in the food supply. The data presented here are important as a benchmark to track changes in TFA intake in the future as the result of these sweeping legislative mandates to decrease TFA in the food supply. It will be important to monitor changes in fatty acid intake in the population because of the concerted effort to remove TFA from the food supply. Much progress has occurred in food science and lipid chemistry to appreciably decrease industrially produced TFA in the food supply in recent years. In fact, a new study reported an average population intake of 1.3 g/day of industrially produced TFA using analysis of 2003–2006 NHANES data [15]. In many instances, oils high in MUFA are replacing conventional fats that are high in TFA (e.g., for liquid fat food applications), whereas in other situations, fat sources of SFA are replacing solid fats rich in TFA (e.g., for solid fat food applications). One caveat to be mindful of is that efforts to decrease TFA should not result in increases in SFA intake in the population. Consistent with this, the American Heart Association [16] has recommended that TFA and SFA in unmodified foods not be greater than total SFA in modified foods. It is clear that there are currently countless fats that differ in their fatty acid profiles that could be substituted for TFA. It will be important that fats devoid of TFA be selected to achieve current dietary fatty acid guidelines and, thereby, realize a public health benefit. This has been reported in a recent modeling exercise by Lefevre et al. [17]. The last point that must be emphasized is to acknowledge that many of the foods that deliver industrially produced TFA are “extras in the diet,” and even if they are modified to have a fatty acid profile that is consistent with current dietary recommendations, their intake should be limited within the context of a healthy diet that meets current food-based and nutrient recommendations. Limiting intake of these fatty acid-modified foods will also help control calories, which is a pressing societal need.

A limitation of this study is that the analysis of TFA intake was based on NHANES 1999–2002 data. The TFA data were derived from the 1995 *Trans* Fatty Acid Database and the USDA Nutrient Database for Standard Reference (Release 15). Consequently, changes that were made in foods in the marketplace (with respect to TFA content) between 1993 (when the TFA analyses of foods were completed) and 1999–2002 are not reflected in this analysis. This is a well-recognized limitation of the available nutrient databases and underscores the importance of continuously updating them. Another potential limitation is the use of two 24-h recalls. According to Allison et al. [11], 3 days of intake data provide a better assessment of usual intake. Moreover, the present study only evaluated industrial TFA and not other sources of TFA, which could have important health implications [18]. Despite these limitations, however, this study provides useful information about TFA intake in the population and important food sources prior to collective efforts by the food industry to decrease TFA in the food supply.

In summary, in all population groups studied, TFA intake between 1999 and 2002 was 5.0–7.8 g/day (2.3–2.7 % of energy). Much of the TFA consumed was derived from energy-dense and nutrient-poor foods such as cakes, cookies, pies, pastries, and savory snacks (i.e., chips). Strikingly, there was a population, albeit small, with an extremely high intake of TFA (males aged 12–19 years in the fifth quintile, who consumed 11.8–92.4 g/day) as well as SFA (and also energy). Obviously, this reflects very poor dietary practices. In fact, as TFA intake increased in the population groups studied, there was a trend toward poorer dietary practices. Ongoing nutrition intervention efforts must be directed at decreasing TFA and also promoting healthy dietary patterns.

## Abbreviations

CSFII: Continuing Survey of Food Intakes by Individuals
FARE: Foods Analysis and Residue Evaluation Program
FDA: U.S. Food and Drug Administration
MUFA: Monounsaturated fatty acids
NHANES: National Health and Nutrition Examination Survey
SFA: Saturated fatty acids
TFA: *Trans* fatty acids
USDA: U.S. Department of Agriculture
